# pH-Dependent Stability and Cooperativity of Protein Unfolding from Differential Scanning Calorimetry Using FitFoldData

**DOI:** 10.3390/biom16091368

**Published:** 2026-09-20

**Authors:** Knarik Yeritsyan, Vladimir Uversky, Artem Badasyan

**Affiliations:** 1 Laboratory of Organic Matter Physics, University of Nova Gorica, Vipavska 13, SI-5000 Nova Gorica, Slovenia; knarik.yeritsyan@ung.si; 2 Department of Molecular Medicine, USF Health Byrd Alzheimer’s Research Institute, Morsani College of Medicine, University of South Florida, 12901 Bruce B. Downs Blvd., MDC07, Tampa, FL 33612, USA; vuversky@usf.edu; 3 Materials Research Laboratory, University of Nova Gorica, Vipavska 13, SI-5000 Nova Gorica, Slovenia

**Keywords:** helix–coil transition, protein folding, pH dependence, hydrogen bonding, Zimm–Bragg model, thermodynamic modeling, DSC data analysis, solvent effects on folding, folding cooperativity, biopolymer stability

## Abstract

Protein folding is highly sensitive to environmental conditions such as pH, which can influence internal hydrogen bonding and interactions with the solvent. In this study, we use our FitFoldData online tool to analyze published differential scanning calorimetry (DSC) data on the unfolding of four proteins measured across different pH values. For each dataset, we examine the fitted thermodynamic parameters, including the intrapeptide and peptide–solvent hydrogen-bonding energies (*h* and $h_{ps}$) and the Zimm–Bragg cooperativity parameter (σ). In the analyzed datasets, under both acidic and alkaline conditions, *h* and $h_{ps}$ range from 2 to 8.5 kJ/mol, while σ is on the order of $10^{-3}$ to $10^{-2}$. In addition, the two-state cooperativity parameter $k_{2}$ is estimated from the DSC curves as the enthalpy ratio. By considering the size-dependent relative fluctuation, $1/\sqrt{N}$, we introduce a “two-state range” around the value $k_{2}=1$ for each protein with a given number *N* of peptide units. We find that pH can shift proteins into or out of the two-state range of $k_{2}$ values.

## 1. Introduction

The helix–coil transition in polypeptides is a fundamental process governing protein folding and stability [1,2,3,4,5]. Thermodynamic approaches such as the Zimm–Bragg framework [6] and its water-incorporating extensions [7,8] have proven effective in describing these transitions [9,10,11,12,13]. However, biological systems operate in complex environments with multiple-component solvents, where solvent conditions, especially pH, can modulate protonation states, hydrogen bonding, and the structure of surrounding water [14,15,16,17,18,19,20,21,22,23]. Moreover, Coulomb interactions between charges is of a long-range nature, and in the case of polyampholytes (such as a polypeptide), a small change in pH may result in complex effects at the level of the whole polymer chain. For instance, enzymes have an optimal pH (like pepsin in the stomach at very low pH or trypsin in the intestine at higher pH). Outside that range, their structure and, therefore, activity decline. Describing the interaction between all the charges present in the system exactly is a complicated and yet fully unresolved topic.

We have chosen a much simpler, yet efficient approach. Building on previous works [7,8], we have developed a helix–coil transition model that incorporates solvent effects and chain-length dependence, enabling accurate fits to differential scanning calorimetry (DSC) data [11]. Within this framework, calorimetric data are characterized by effective thermodynamic parameters, including the intrapeptide and peptide–solvent hydrogen bonding energies (*h* and $h_{ps}$) and the cooperativity parameter (σ), which describes how much more difficult the helix initiation is compared to helix growth. In the context of our study, the model and its fitted parameters should be interpreted as effective macroscopic thermodynamic descriptors of global unfolding, rather than as strictly microscopic single-helix parameters. Although the model does not explicitly include pH as a variable, these fitted descriptors implicitly reflect the influence of solvent conditions on folding thermodynamics.

Protein thermal stability can be characterized using complementary methods ranging from conventional DSC to differential scanning fluorimetry and proteome-scale thermal profiling [24,25,26,27]. Modern analyses also emphasize that the thermodynamic interpretation of unfolding curves depends on reversibility, scan protocol, and the selected unfolding model [28,29,30,31]. Recent DSC studies further illustrate the sensitivity of protein stability to solvent composition and dissolved additives [32,33].

Protein structure and stability are strongly influenced by pH because protonation or deprotonation of ionizable groups changes net charge and electrostatic interactions, thereby shifting the balance between native and non-native conformations [34,35]. When pH is moved far from the range that favors the native fold, many proteins denature and may populate conformations ranging from compact partially folded intermediates to highly unfolded ensembles [34,35,36,37,38]. One well-established class of partially folded intermediate is the molten globule, which retains substantial secondary structure but lacks tight native tertiary packing [37,39]. Under such strongly destabilizing conditions, thermal unfolding need not remain an ideal cooperative two-state process, and DSC endotherms can broaden or reflect predenaturational and other non-native conformational changes rather than a single native-to-unfolded transition [35,40]. Accordingly, DSC data recorded at strongly destabilizing pH values should be interpreted with particular caution [40].

In the present work, we use a previously established framework, implemented in the FitFoldData web tool [41], to examine how the corresponding thermodynamic parameters vary with pH. Using published DSC datasets for several proteins measured across different pH values, we analyze the pH dependence of the model-derived quantities. Some of these parameter values were also reported in our earlier DSC-focused study [11]; here, however, they are examined specifically from the perspective of pH-dependent behavior across different protein systems. The novelty of the present work therefore lies not in the formulation of the model or the fitting procedure, but in the comparative analysis of how these thermodynamic descriptors behave as a function of pH.

**Figure 1:**
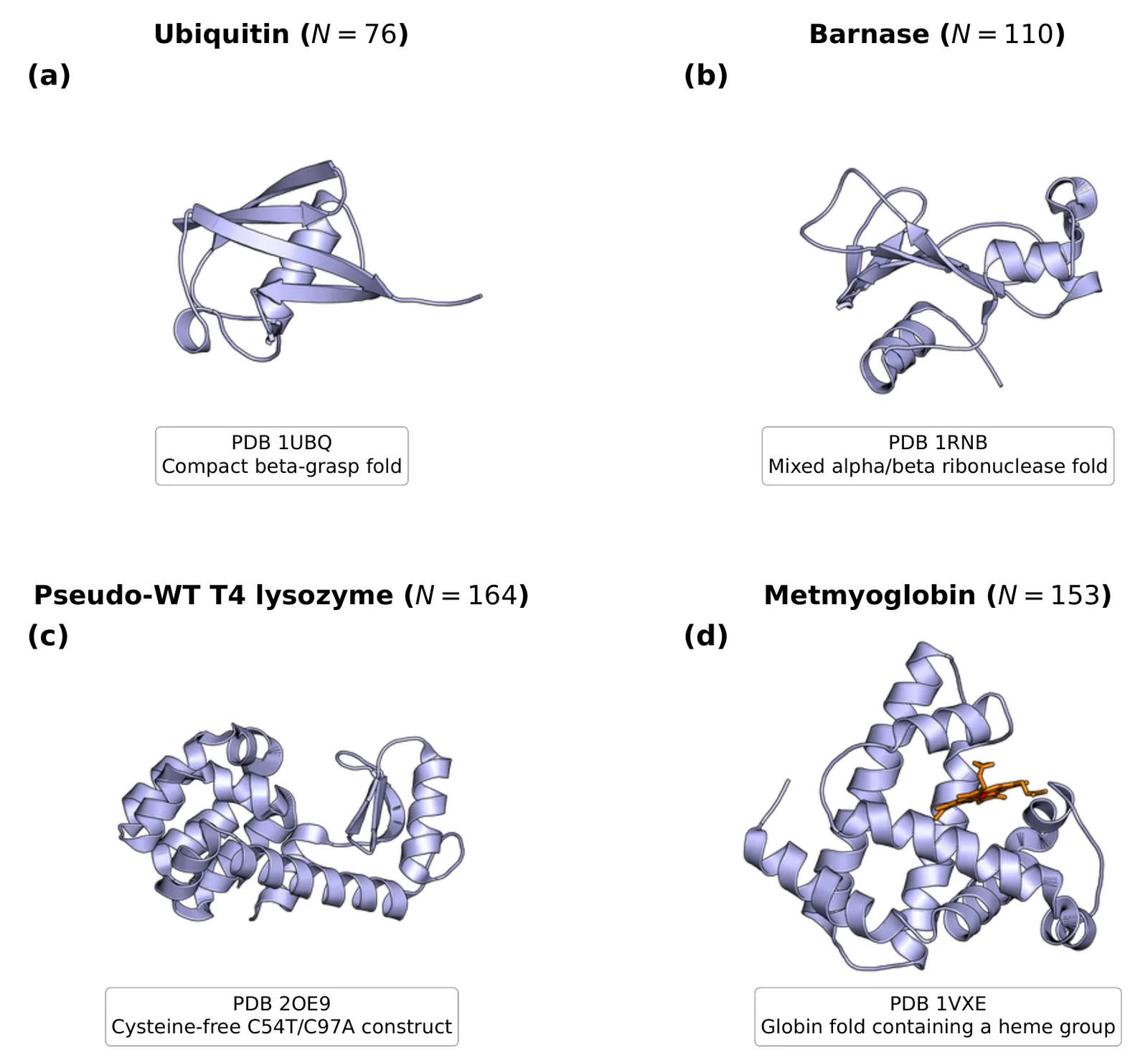
Representative experimental structures of the four proteins analyzed in this study: (**a**) ubiquitin (N=76; PDB 1UBQ), (**b**) barnase (N=110; PDB 1RNB), (**c**) pseudo-WT T4 lysozyme (N=164; PDB 2OE9), and (**d**) sperm whale metmyoglobin (N=153; PDB 1VXE). The pseudo-WT T4 lysozyme construct contains the cysteine-removing substitutions C54T and C97A. These PDB structures are shown solely to illustrate the overall protein architectures; they were not necessarily determined at the pH values or under the solution conditions used for the analyzed DSC measurements and should not be interpreted as structures adopted under those experimental conditions.

The four proteins considered in this study differ substantially in size and architecture (Figure 1). Ubiquitin is a compact 76-residue protein with a beta-grasp fold, barnase is a 110-residue alpha/beta ribonuclease, T4 lysozyme is a 164-residue two-lobed protein, and metmyoglobin is a 153-residue heme-containing globular protein. These representative structures provide structural context for comparing their pH-dependent thermal behavior.

By comparing the behavior of *h*, $h_{ps}$, their difference ($h-h_{ps}$), the transition temperature ($T_{m}$), and the cooperativity parameter σ across different proteins and pH conditions, we aim to distinguish shared trends from protein-specific responses. This analysis provides insight into how environmental factors modulate folding stability and cooperativity, and highlights how model-derived thermodynamic quantities can be used to probe pH-dependent effects in protein systems.

## 2. Methods

The theoretical framework used in this work is the previously established Zimm–Bragg model [6] in water [7,8,11]. Within the framework, calorimetric data are characterized by the fitted parameters $t_{0}$, *h*, $h_{ps}$, and *Q*, where $t_{0}$ is the glass transition temperature, *h* and $h_{ps}$ represent the effective intrapeptide and peptide–solvent hydrogen bonding energies, respectively; the parameter *Q* reflects the entropic cost of helix formation and is related to the Zimm–Bragg cooperativity parameter σ=1/Q. The cooperativity parameter σ determines the sharpness of the helix–coil transition: smaller values of σ correspond to higher cooperativity and sharper transitions, while larger values indicate broader, less cooperative transitions. In the present work, we focus on the pH dependence of *h*, $h_{ps}$, σ, and the melting temperature $T_{m}$, defined as the temperature of the DSC peak maximum. We use the FitFoldData web tool [41] and process several published DSC datasets measured at different pH values.

The covariance matrix returned by the nonlinear least-squares fit was used to estimate the standard uncertainties, covariances, and correlation coefficients of the fitted parameters. Because *h* and $h_{ps}$ were determined simultaneously from the same fit, the standard uncertainty of their difference was calculated by first-order uncertainty propagation including their covariance [42]. According to the law of propagation of uncertainty, the combined standard uncertainty of $h-h_{ps}$ is

(1) $$\begin{array}{cc} u_{c}^{2}\left(h-h_{ps}\right) & =u^{2}\left(h\right)+u^{2}\left(h_{ps}\right)-2u\left(h,h_{ps}\right)\\ \; & =u^{2}\left(h\right)+u^{2}\left(h_{ps}\right)-2r\left(h,h_{ps}\right)u\left(h\right)u\left(h_{ps}\right), \end{array}$$

where u(h) and $u(h_{ps})$ are the standard uncertainties of the fitted parameters, $u(h,h_{ps})$ is their covariance, and $r(h,h_{ps})$ is their correlation coefficient, defined as

(2) $$r\left(h,h_{ps}\right)=\frac{u(h,h_{ps})}{u\left(h\right)u\left(h_{ps}\right)}.$$

The cooperativity parameter was calculated as σ=1/Q. Its combined standard uncertainty was obtained by first-order propagation:

(3) $$u_{c}\left(\sigma \right)=\left|\frac{\partial \sigma }{\partial Q}\right|u\left(Q\right)=\frac{u(Q)}{Q^{2}},$$

where u(Q) is the standard uncertainty of the fitted parameter *Q*. The reported error bars represent approximate standard uncertainties (k=1) obtained from the local covariance matrix of the nonlinear least-squares fit.

The underlying experimental datasets were taken from previously published studies on ubiquitin, barnase, pseudo-WT T4 lysozyme, and metmyoglobin [40,43,44].

For each protein and pH value, we considered the fitted thermodynamic parameters *h*, $h_{ps}$, and σ, and additionally analyzed the quantity $h-h_{ps}$ as a measure of the balance between internal and solvent-mediated stabilization. The purpose of the present study is therefore not to introduce a new fitting model, but to examine how these thermodynamic descriptors vary systematically with pH across different protein systems.

**Figure 2:**
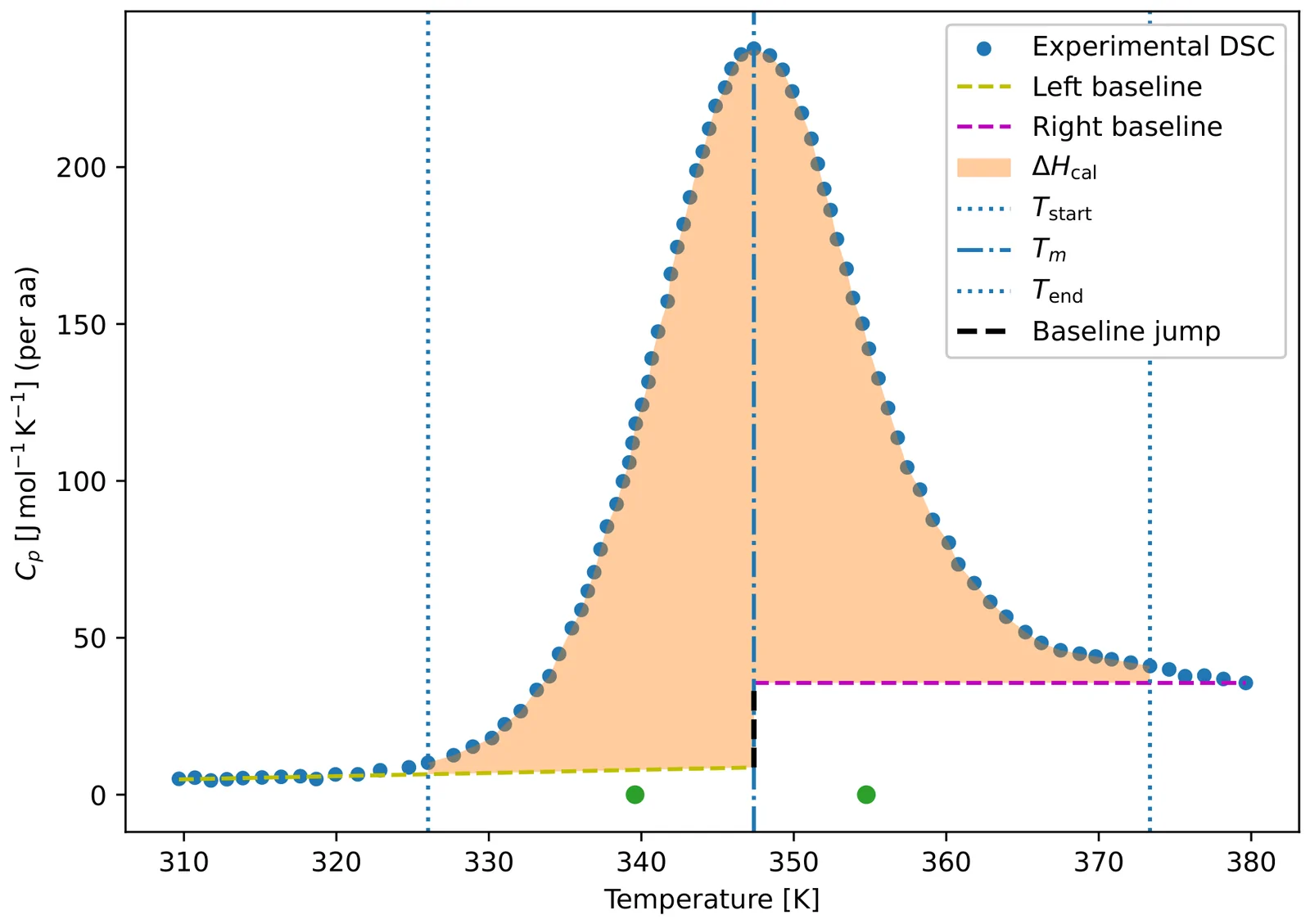
Differential scanning calorimetry (DSC) curve and determination of the calorimetric enthalpy. Experimental heat-capacity data ($C_{p}$) are shown together with the constructed baselines. The left baseline (low-temperature side) is obtained by linear regression of selected pre-transition heat-capacity points, as described in the Methods. The right baseline (high-temperature side) is taken as a constant equal to the heat capacity at the highest measured temperature. These two baselines are connected at the peak position, defining a baseline jump at $T_{m}$, the temperature of the maximum of the DSC peak. The onset ($T_{\mathrm{start}}$) and end ($T_{\mathrm{end}}$) of the transition are defined as the temperatures where the baseline-corrected heat capacity reaches 2% of the peak height on the low- and high-temperature sides, respectively. The shaded area between the experimental curve and the baseline corresponds to the calorimetric enthalpy $\Delta H_{\mathrm{cal}}$. The green points indicate the temperatures corresponding to half of the maximum excess heat capacity ($\Delta C_{p}/2$), used to determine the transition width $\Delta T_{1/2}$.

Additionally, we monitor the two-state folding cooperativity criterion [9,45]:

(4) $$k_{2}=\frac{\Delta H_{vH}}{\Delta H_{cal}},$$

the ratio of the van’t Hoff enthalpy $\Delta H_{vH}=\frac{4RT_{m}^{2}}{\Delta T_{1/2}}$ to the calorimetric enthalpy $\Delta H_{cal}=\int_{T_{start}}^{T_{end}}C_{p}\left(T\right)dT$. Here, $T_{m}$ is the transition temperature, and $\Delta T_{1/2}$ is the full width at half maximum of the excess heat capacity peak, and the calorimetric enthalpy $\Delta H_{cal}$ is obtained by numerical integration of the baseline-corrected excess heat capacity. $k_{2}$ helps to define the size of the cooperative unit (the number of subunits or amino acid residues that act as a single unit during a transition) and is equal to unity in the case of the two-state transition. Both $\Delta H_{cal}$ and $\Delta H_{vH}$ are found from digitized DSC curves using a consistent baseline construction and transition width definition, as shown in Figure 2. In particular, the figure shows the construction of the baselines, the definition of the transition region ($T_{start}-T_{end}$), and the extraction of both the calorimetric enthalpy $\Delta H_{cal}$ and the transition width $\Delta T_{1/2}$ used for estimating $\Delta H_{vH}$. For construction of the low-temperature baseline, points within the lowest 20% of the smoothed pre-transition heat-capacity values were selected. If fewer than six points satisfied this criterion, the first 30% of the pre-transition data points were used instead. Smoothing was applied only for point selection and determination of the transition geometry; the linear baseline itself was fitted to the original, unsmoothed heat-capacity data. The high-temperature baseline was defined as a constant equal to the heat capacity at the final measured temperature. Obtained values are sensitive to baseline choice, smoothing, and digitization accuracy [11].

## 3. Results and Discussion

**Figure 3:**
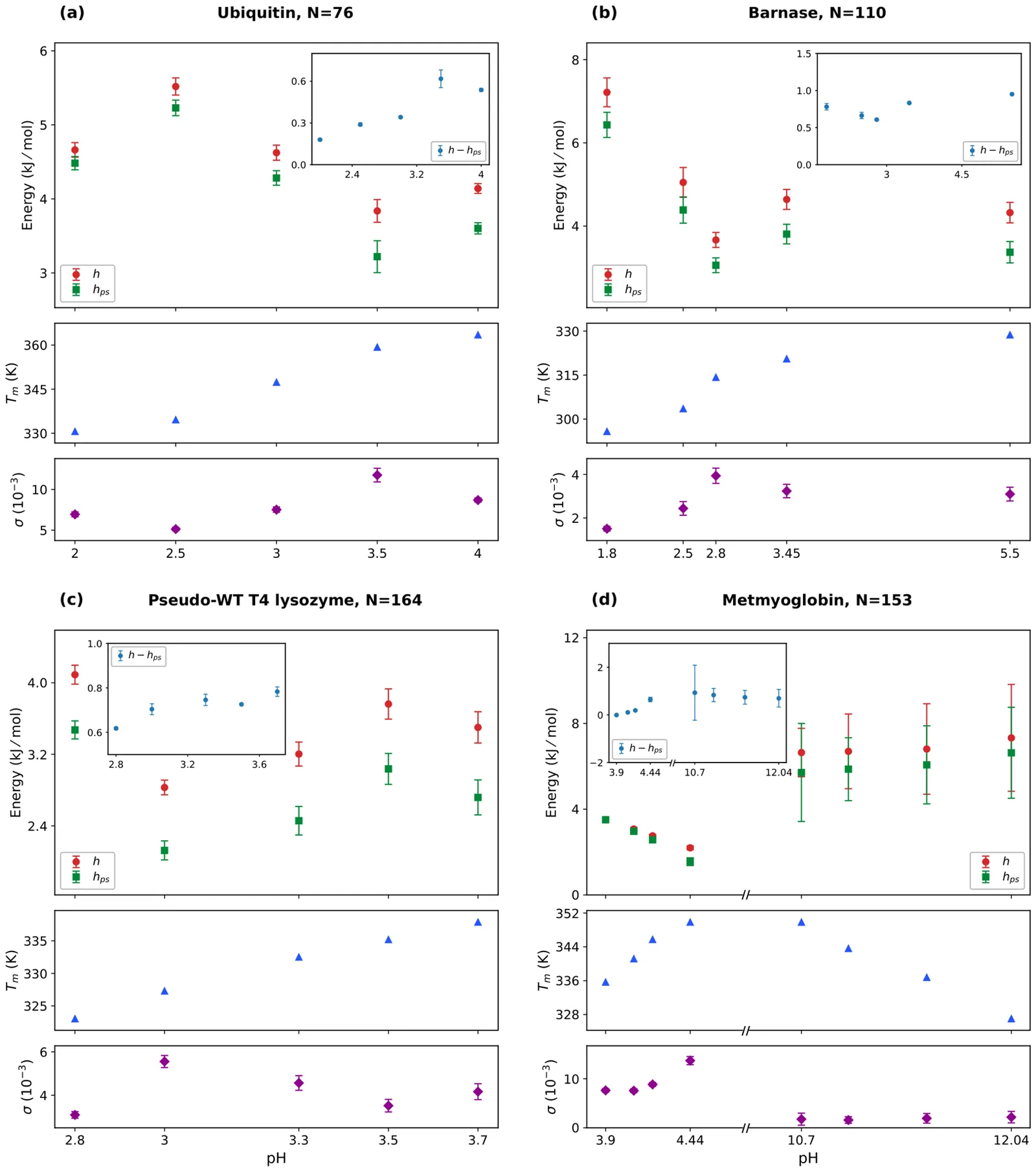
pH dependence of thermodynamic parameters for (**a**) ubiquitin (N=76), (**b**) barnase (N=110), (**c**) pseudo-WT T4 lysozyme (N=164), (**d**) metmyoglobin (N=153). Values of h, $h_{\mathrm{ps}}$, and σ are based on analysis of DSC data from (**a**) Figure 1b of Ref. [40], (**b**) Figure 1a of Ref. [40], (**c**) Figure 2 of Ref. [43], (**d**) Figure 3a,c of Ref. [44]. h and $h_{\mathrm{ps}}$ are intrapeptide and peptide–solvent hydrogen bonding parameters. Insets show $h-h_{\mathrm{ps}}$ vs. pH. $T_{m}$ is the melting temperature, and σ=1/Q is the cooperativity parameter. Inset error bars represent one-standard-deviation uncertainties propagated from the fitted covariance matrix, including the correlation between h and $h_{\mathrm{ps}}$.

Figure 3 summarizes the pH dependence of thermodynamic parameters for four proteins: ubiquitin and barnase [40], pseudo-WT T4 lysozyme [43], and sperm whale metmyoglobin [44].

We characterize protein stability using the melting temperature ($T_{m}$), together with the intrapeptide and peptide–solvent hydrogen bonding parameters (*h* and $h_{ps}$), their difference ($h-h_{ps}$), and the cooperativity parameter (σ=1/Q). Across the four proteins studied, $T_{m}$ and $h-h_{ps}$ exhibit a consistent pH dependence, whereas the behavior of σ is more protein-specific.

**Hydrogen Bonding Parameters (**h **and** $h_{\mathrm{ps}}$ **)**

Across all proteins, both intrapeptide (h) and peptide–solvent ($h_{\mathrm{ps}}$) hydrogen bonding parameters exhibit notable pH dependence. In general, $h_{\mathrm{ps}}$ is larger at lower pH, whereas the difference $h-h_{\mathrm{ps}}$ increases toward higher pH. Within the fitted thermodynamic description, this behavior is consistent with stronger relative peptide–solvent stabilization under acidic conditions and a progressive shift toward internal stabilization at higher pH.

To quantify this balance, we analyze the difference $h-h_{\mathrm{ps}}$, shown in the insets of Figure 3. For all proteins studied, $h-h_{\mathrm{ps}}$ increases monotonically with pH, within the acidic-to-mildly-acidic range. In the case of metmyoglobin, however, the extended pH dataset reveals deviations from this behavior at strongly alkaline conditions.

**Melting temperature ($T_{m}$)**

Across all datasets within the acidic-to-mildly-acidic range, the melting temperature $T_{m}$ increases with pH. For metmyoglobin, however, the extended dataset (see the middle panels of Figure 3) shows that this trend breaks down at extreme pH and indicates the shift of thermal midpoint of unfolding to higher temperatures under progressively less acidic conditions (up to the maximum measured pH of 5.5). The increasing $T_{m}$ is consistent with the observed rise of $h-h_{\mathrm{ps}}$, suggesting that conditions which more strongly favor internal stabilization relative to peptide–solvent interactions also increase the thermal stability of the folded state.

**Cooperativity Parameter (σ)**

In the Zimm–Bragg model, the stability parameter s=exp[−ΔG/RT] denotes the statistical weight for helix growth, whereas helix initiation carries a statistical weight of σs. The factor σ≪1 reflects the unfavorable free-energy cost of nucleating a helical region in a predominantly coil environment.

The pH dependence of σ reveals complex, protein-specific trends. These variations underscore the importance of sequence and fold topology in determining the sensitivity of folding cooperativity to environmental changes. The different trends observed for metmyoglobin and T4 lysozyme (see Figure 3), for example, suggest that the pH response of cooperativity depends on protein-specific structural constraints and solvent exposure.

Together, these observations show that pH, although not explicitly included in the model, leaves measurable signatures in the fitted thermodynamic descriptors. The present comparative analysis highlights how model-derived quantities can be used to examine pH-dependent changes in folding stability and cooperativity, and may be useful for understanding environmentally modulated conformational transitions.

The clear distinction in the behavior of different proteins—ubiquitin, barnase, T4 lysozyme, and metmyoglobin—across pH ranges confirms that sequence-specific factors and structural constraints influence how hydrogen bonding and cooperativity respond to solvent conditions. While some trends, such as the increase of $T_{m}$ and $h-h_{\mathrm{ps}}$ with pH, are common across all systems studied, the detailed response of σ is more strongly dependent on protein-specific structural features.

The pH-dependent trends in these quantities underscore the complex interplay between protonation, hydrogen bonding, and solvent structuring in helix–coil transitions. In particular, the combined behavior of $T_{m}$ and $h-h_{\mathrm{ps}}$ provides a consistent picture of pH-dependent stability within the measured range: increasing pH across the acidic-to-mildly-acidic regime is associated with stronger relative internal stabilization and higher thermal stability.

### 3.1. Physicochemical Mechanisms Underlying pH Dependence in the Acidic Regime

While most analyzed datasets extend only up to pH 5.5, additional data for metmyoglobin provide insight into behavior at higher pH. These results indicate that the trends observed in the acidic regime do not extend to extreme pH conditions, and that protein stability exhibits a non-monotonic dependence on pH. Accordingly, the increase of $T_{m}$ and $h-h_{\mathrm{ps}}$ with pH is robust only within the acidic-to-mildly-acidic range and should not be extrapolated to neutral or alkaline conditions. We therefore interpret our results as describing how stability and hydrogen-bond balance evolve within this regime, while extension to physiological pH requires targeted DSC measurements or complementary titration experiments.

All proteins studied here have mature-sequence theoretical isoelectric points (pI) in the near-neutral-to-basic range (approximately pH 6.6–9.6) [46,47,48,49]. Therefore, the acidic subsets of the DSC datasets lie below the corresponding pI values, so the proteins are expected to carry a net positive charge under those conditions, which may influence solvent interactions and the balance between intrapeptide and peptide–solvent hydrogen bonding.

The theoretical isoelectric point (pI) is the specific pH at which a protein carries a net electrical charge of zero, balancing its positive and negative amino acid charges. This value is essential because a net charge of a protein dictates its solubility, structure, and molecular interactions at any given pH. In environments more acidic than its pI (lower pH), a protein gains protons and becomes positively charged, whereas in more basic environments (higher pH), it loses protons and becomes negatively charged. Based on their amino acid sequences, the theoretical isoelectric points (pI) of four proteins analyzed in this study (as given by ProtParam https://web.expasy.org/protparam/ (accessed on 30 August 2026)) are: barnase (pI 9.30), T4 lysozyme (pI 9.59), myoglobin (pI 8.70), and ubiquitin (pI 6.56). Based on their theoretical isoelectric points (pI), these four proteins carry different net surface charges depending on the pH of their surrounding environment: at acidic pH (below pH 5.0), all four proteins are expected to be positively charged; at neutral pH (7.0), barnase, T4 lysozyme, and myoglobin are positively charged, whereas ubiquitin is negatively charged; and at basic pH (9.0), barnase and T4 lysozyme are positively charged, and myoglobin and ubiquitin are negatively charged. Protein stability and unfolding are directly influenced by pI-driven pH responses. Environmental pH directly alters the ionization states of amino acid side chains, such as the carboxyl groups on aspartate/glutamate and amino groups on lysine/arginine. When these groups gain or lose charges, vital salt bridges and hydrogen bonds that lock the native conformation in place are broken. As the pH deviates significantly from a protein’s pI, the molecule accumulates a high density of net positive or negative charges. The resulting severe intramolecular electrostatic repulsion destabilizes the tertiary structure, disrupts the native electrostatic networks and triggers denaturation and unfolding.

The structural stability of the protein across the investigated pH range is heavily governed by the ionization states of acidic amino acid side chains, particularly aspartate (Asp, pKa ∼ 3.9) and glutamate (Glu, pKa ∼ 4.3). At the lowest investigated pH (∼2.0), these carboxylate groups undergo complete protonation, neutralizing their inherent negative charges. This selective loss of negative charge disrupts existing salt bridges and eliminates local electrostatic balance. Consequently, the unshielded positive charges on basic residues (Lys (pKa ∼ 10.5), Arg (pKa ∼ 13.8), His (pKa ∼ 6.0)) experience severe electrostatic repulsion. This intra-chain repulsion destabilizes the native fold, driving global acid-induced unfolding. The structural consequences of this acid-induced destabilization are directly reflected in the fitted effective parameters, most notably the elevation of the parameter hps that serves as a direct indicator of backbone solvent exposure. The marked increase of this parameter at pH 2.0 serves as a direct indicator of structural expansion. As electrostatic repulsion forces the backbone to unravel, tightly packed core segments become solvent-accessible. This increases the total solvent-exposed surface area (SASA), maximizing the geometric opportunities for water molecules to form hydrogen bonds with the peptide backbone. Concurrently, the disruption of native α-helices and β-sheets causes a significant decline in the intrapeptide hydrogen-bonding parameter h, as local secondary structures are systematically broken to accommodate chain expansion. Consequently, the net parameter ($h-h_{\mathrm{ps}}$) reaches its minimum, or becomes significantly negative, reflecting a thermodynamic regime where peptide-solvent interactions energetically outcompete self-association, driving the forward unfolding equilibrium. On the other hand, increasing the pH toward the 4.0–5.5 range initiates a progressive deprotonation of the Asp and Glu carboxyl groups. The progressive deprotonation of these carboxyl groups introduces localized negative charges back into the matrix. This charge restoration immediately mitigates the global electrostatic repulsion among basic residues. More importantly, it facilitates the spatial pairing of oppositely charged side chains, enabling the formation of stabilizing salt bridges and favorable Coulombic networks. The relief of electrostatic stress and the emergence of salt bridges shift the effective parameters in the opposite direction. The polypeptide chain collapses back into a more compact, native-like topology, shielding the backbone from the aqueous environment. This structural compaction is manifested by a sharp decrease in $h_{\mathrm{ps}}$ due to reduced solvent exposure, and a reciprocal rise in h as stable, native intrapeptide hydrogen-bonding networks reform. The resulting increase in the net parameter ($h-h_{\mathrm{ps}}$) provides a clear, parameter-based quantification of the thermodynamic stabilization achieved through side-chain ionization control. By tracking these shifts, the parameters h, $h_{\mathrm{ps}}$, and their difference ($h-h_{\mathrm{ps}}$) serve as sensitive macroscopic reporters of microscopic side-chain ionization events and the structural transitions characteristic of acid-induced unfolding.

### 3.2. Physical Origin of Metmyoglobin Destabilization Under Alkaline Conditions

For metmyoglobin, the datasets from different pH ranges were combined in Figure 3d to examine the behavior over a wider pH interval. In the acidic-to-mildly-acidic region, $T_{m}$ and $h-h_{\mathrm{ps}}$ increase with pH, consistent with the trends observed for the other proteins. However, at strongly alkaline pH, this trend is reversed: $T_{m}$ decreases and $h-h_{\mathrm{ps}}$ no longer increases, indicating reduced internal stabilization and loss of cooperative folding behavior. This shows that the monotonic increase observed in the acidic regime should not be extrapolated to extreme pH conditions.

The drastic reduction in thermal transition temperature (Tm) and loss of unfolding cooperativity of myoglobin under extreme alkaline conditions (pH 10.7 and 12.04) diverge fundamentally from acidic denaturation mechanisms. Above pH 10.0, the deprotonation of tyrosine (pKa ∼ 10.1) and lysine (pKa ∼ 10.5) generates severe polyanionic electrostatic repulsion within the protein matrix. This structural stress alters the heme pocket geometry and forces a change in heme coordination, ultimately driving heme dissociation to form unstable apo-myoglobin. Bereft of its stabilizing cofactor, the protein denatures rapidly, shifting from a cooperative, two-state unfolding mechanism to a pathway dominated by the presence of molten globule-like intermediate. In fact, Postnikova et al. [50] indicated that at alkaline pH (pH 12.5–13), myoglobin exists as an alkaline-denatured state characterized by the lack of unique spatial structure, combined with the presence of ∼80% of helical structure and noticeable compactness. This alkaline pH-induced structural destabilization explains the large errors in Figure 3d, incomparable with other proteins considered. Whether this is a unique case, or a general behavior for many proteins, is unclear due to the unavailability of high-pH experimental data for other proteins.

### 3.3. Two-State Cooperativity Parameter $k_{2}$

The parameter $k_{2}=\Delta H_{\mathrm{vH}}/\Delta H_{\mathrm{cal}}$ (see Equation (4) and the following paragraph) has several interesting features that should be discussed before we proceed to our results.

Proteins consist of finite polypeptide chains, typically containing tens to hundreds of amino acid residues, and finite-size effects are therefore inherent to their thermodynamic behavior. It is consequently useful to discuss the relevant thermodynamic quantities without invoking a macroscopic phase transition or an idealized two-state picture. Experimentally, a DSC thermogram of a finite protein system, such as that shown in Figure 2, exhibits a finite and broadened transition rather than a true singularity in the heat capacity. In addition to finite-size effects, the shape and width of the transition may reflect the heteropolymeric nature of natural polypeptides and their quasi-one-dimensional structural organization. Accordingly, the systems considered here contain a finite number of interacting units and should not be regarded as undergoing a macroscopic phase transition.

Before using the enthalpy ratio as a criterion for two-state folding, an important caveat should be emphasized. As discussed in detail in Ref. [9] (see the paragraph following Equation (18)), the condition $k_{2}=1$ is necessary but not sufficient for two-state behavior. Thus, a genuine two-state folder is expected to exhibit a value of $k_{2}$ close to unity, whereas the observation of $k_{2}\approx 1$ alone does not establish a two-state folding mechanism. The two-state character of a protein should therefore be assessed using $k_{2}$ together with complementary thermodynamic and structural evidence [51].

The values of $k_{2}=\Delta H_{\mathrm{vH}}/\Delta H_{\mathrm{cal}}$ reported here were obtained from our own numerical analysis of the digitized DSC curves rather than taken directly from tabulated literature values. The procedure used to determine both the calorimetric and van’t Hoff enthalpies from the DSC curves is illustrated in Figure 2. The figure shows the baseline construction, the definition of the transition interval ($T_{\mathrm{start}}-T_{\mathrm{end}}$), and the extraction of the calorimetric enthalpy $\Delta H_{\mathrm{cal}}$ together with the transition half-width $\Delta T_{1/2}$ used to estimate $\Delta H_{\mathrm{vH}}$.

Because the ratio $\Delta H_{\mathrm{vH}}/\Delta H_{\mathrm{cal}}$ is sensitive to baseline construction, smoothing, digitization accuracy, and the definition of the transition width, small deviations from unity are expected even for systems that closely approximate two-state behavior. Consequently, the values reported here should be regarded as indicators of the apparent cooperativity of the folding transition.

**Figure 4:**
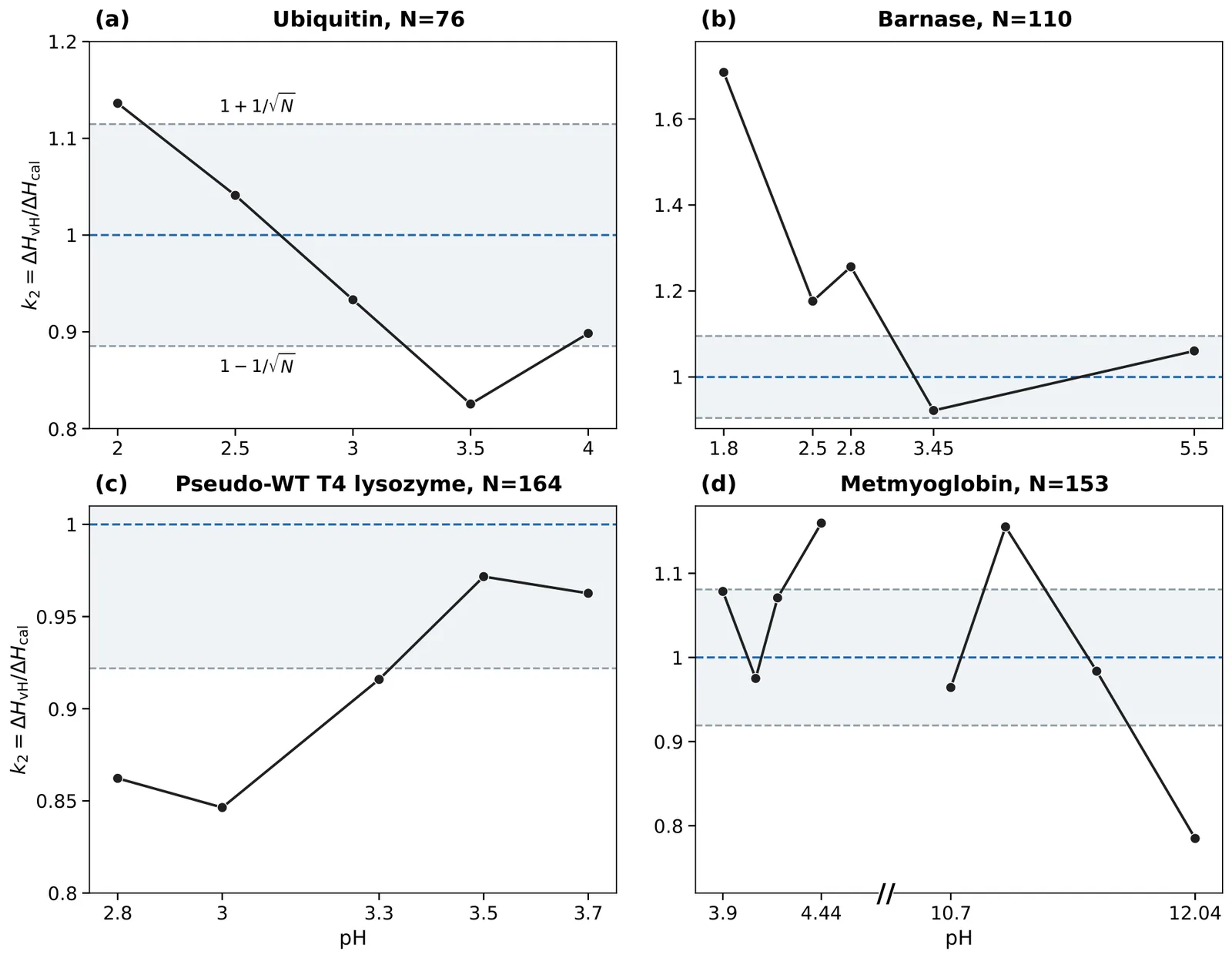
Ratios of van’t Hoff to calorimetric enthalpy ($k_{2}=\Delta H_{\mathrm{vH}}/\Delta H_{\mathrm{cal}}$) as a function of pH for ubiquitin, barnase, pseudo-WT T4 lysozyme, and metmyoglobin. The combined acidic and alkaline pH datasets are shown for metmyoglobin, extending the accessible pH range. The ratio provides an estimate of apparent cooperativity, where values close to unity indicate approximately two-state behavior, while deviations may reflect non-ideal or multi-state transitions as well as sensitivity to baseline construction, digitization, and transition-width determination. Dashed gray lines indicate the borders of the “two-state interval” (shaded in gray) around the $k_{2}=1$ value: $1-1/\sqrt{N},1+1/\sqrt{N}$. Dashed blue lines indicate the $k_{2}=1$ value. Black dots represent the calculated $k_{2}$ values, while black lines connecting them serve as a guide to the eye.

Having said all the above, we can now proceed to the resulting values of $\Delta H_{\mathrm{vH}}/\Delta H_{\mathrm{cal}}$ as a function of pH for all proteins considered in Figure 4. The figure demonstrates systematic deviations from ideal two-state behavior and illustrates the dependence on pH.

Although the influence of pH on $k_{2}$ is evident, two important questions remain. First, how large a deviation from unity can still be regarded as consistent with two-state behavior? Second, is the acceptable range of $k_{2}$ around unity universal, or does it depend on the size of the protein? To address these questions, we return to a fundamental result of Statistical Mechanics: the relative fluctuations of thermodynamic quantities (not in the immediate vicinity of a phase transition point) are of the order of $1/\sqrt{N}$, where N is the number of particles in the system [52]. In the present context, N corresponds to the number of repeated units, i.e., amino acid residues. Assuming that the relative finite-size fluctuations of the two enthalpic quantities entering the ratio remain of order $1/\sqrt{N}$, and that their correlations do not alter this scaling, the relative uncertainty of their ratio is likewise expected to be of order $1/\sqrt{N}$.

This observation suggests replacing the idealized criterion $k_{2}=1$ with a finite-size interval extending from $1-\frac{1}{\sqrt{N}}$ to $1+\frac{1}{\sqrt{N}}$. There are questions that may be addressed to the estimate of relative fluctuations for a particular physical phenomenon, but since the exact classification of a protein folding in terms of Physics is not at hand, the estimate of a statistical uncertainty for $k_{2}$ we propose should be good enough as a starting point or a rule of a thumb.

Throughout this work, we refer to this interval as the “two-state range,” and it is highlighted by the shaded regions in Figure 4. An immediate consequence is that smaller proteins possess a broader admissible two-state range than larger proteins. This observation also raises an interesting question: could finite-size effects partly explain why small proteins are more frequently classified as two-state folders?

This interpretation is broadly consistent with the experimental observations. Ubiquitin (N=76, Figure 4a), the smallest protein examined, lies within the proposed two-state range over nearly the entire pH interval investigated. Barnase (N=110, Figure 4b), the second smallest protein, deviates substantially from two-state behavior at low pH but enters the proposed two-state range above approximately pH 3. Pseudo-WT T4 lysozyme (N=164, Figure 4c), the largest protein in this study, falls within the two-state range only for pH values above approximately 3.3 and exhibits clear non-two-state behavior below this threshold. Metmyoglobin (N=153, Figure 4d) remains within the proposed two-state range over much of the investigated pH interval, although it departs from this range at several pH values. Interestingly, as discussed above, under strongly alkaline conditions, metmyoglobin is believed to deviate substantially from the compact globular state and from two-state behavior. In this regard, it is important to reiterate that, as demonstrated theoretically in Ref. [9], a protein exhibiting a strictly two-state distribution of conformational states must satisfy the condition $k_{2}=1$. However, there is no theoretical basis for the inverse implication; namely, observing $k_{2}=1$ alone does not establish that the protein follows a two-state folding mechanism. Conventionally, values of $k_{2}<1$ are interpreted as indicating reduced calorimetric cooperativity, often associated with the population of unfolding intermediates or multistate transitions. In contrast, values of $k_{2}>1$ may indicate intermolecular association, oligomerization, or other concentration- dependent processes accompanying the thermal transition [51,53].

In order to avoid possible misunderstanding regarding the calorimetric cooperativity of lysozyme in view of Figure 4, let us provide some clarification. At first glance, there appears to be a discrepancy between the classic calorimetric study of hen egg-white lysozyme by Peter L. Privalov and N. N. Khechinashvili (1974) [54], which reported a calorimetric cooperativity ratio close to unity and interpreted unfolding as a highly cooperative two-state transition, and the later DSC study of T4 lysozyme by Carra, Murphy, and Privalov (1996) [43], which reported substantially lower cooperativity ratios for several mutants. In calculating $\Delta H_{\mathrm{vH}}/\Delta H_{\mathrm{cal}}$ shown in Figure 4, we used the data from the last one. This apparent paradox is resolved by recognizing that the studies concern different proteins and different structural contexts. Whereas hen egg-white lysozyme behaves as a single highly cooperative unfolding unit under the conditions examined, the T4 lysozyme mutants exhibit reduced cooperativity and partial population of intermediate states. As noted by Carra, Murphy, and Privalov (1996) [43], “*The $\Delta H_{\mathrm{fit}}$-to-$\Delta H_{\mathrm{cal}}$ ratios of the mutant proteins are significantly less than 1 (Table 1), indicating reduced cooperativity*”. Thus, a cooperativity ratio near unity is not a universal property of all lysozymes but rather reflects the extent to which a particular protein unfolds as a single cooperative unit.

## 4. Conclusions

In this work, using the online tool FitFoldData, developed by us earlier, we analyzed the pH dependence of key thermodynamic descriptors of protein folding using a previously established helix–coil transition model and published DSC data for several proteins. Although the model does not explicitly include pH, the resulting hydrogen bonding energies h, $h_{\mathrm{ps}}$, their difference ($h-h_{\mathrm{ps}}$), melting temperature $T_{m}$, and cooperativity parameter σ reveal systematic and physically interpretable trends across the measured pH range.

In particular, the consistent increase of $T_{m}$ and $h-h_{\mathrm{ps}}$ with pH observed across all proteins within the acidic-to-mildly-acidic regime indicates a progressive shift toward stronger relative internal stabilization under less acidic conditions. However, analysis of additional datasets for metmyoglobin at higher pH demonstrates that this trend does not extend to strongly alkaline conditions, where both $T_{m}$ and $h-h_{\mathrm{ps}}$ decrease, indicating loss of internal stabilization and reduced folding cooperativity. This behavior is consistent with pH-induced denaturation at extreme conditions.

We have extended our analysis for the calculation of $k_{2}=\Delta H_{\mathrm{vH}}/\Delta H_{\mathrm{cal}}$ two-state cooperativity parameter and have shown its pH-dependence. Interestingly, when size-dependent relative fluctuation is taken into account, the otherwise undefined “two-state region” around $k_{2}=1$ becomes quantified.

## Data Availability

No new data were created or analyzed in this study. Data sharing is not applicable to this article.
